# Supplementary material for: Achieving near-perfect light absorption in atomically thin transition metal dichalcogenides through band nesting
Source: Nat Commun. 2023 Jul 1;14:3889. doi: 10.1038/s41467-023-39450-0 (PMC10314950; doi:10.1038/s41467-023-39450-0)
Supplement: Supplementary file 1 — Supplementary Information [file 41467_2023_39450_MOESM1_ESM.pdf]

Supporting Information

Achieving near-perfect light absorption in atomically thin transition metal dichalcogenides through band nesting

Seungjun Lee,[1,*] Dongjea Seo,[1,*] Sang Hyun Park,[1] Nezhueytl Izquierdo,[1] Eng Hock Lee,[1] Rehan Younas,[2] Guanyu Zhou,[2] Milan Palei,[2] Anthony J. Hoffman,[2] Min Seok Jang,[3] Christopher L. Hinkle,[2] Steven J. Koester,[1,†] and Tony Low[1,4,‡]

[1]*Department of Electrical and Computer Engineering, University of Minnesota, Minneapolis, MN 55455, USA*

[2]*Department of Electrical Engineering, University of Notre Dame, Notre Dame, IN 46556, USA*

[3]*School of Electrical Engineering, Korea Advanced Institute of Science and Technology, Daejeon 34141, Republic of Korea*

[4]*School of Physics and Astronomy, University of Minnesota, Minneapolis, MN 55455, USA*

(Dated: June 21, 2023)

S1. TRANSFER MATRIX METHOD

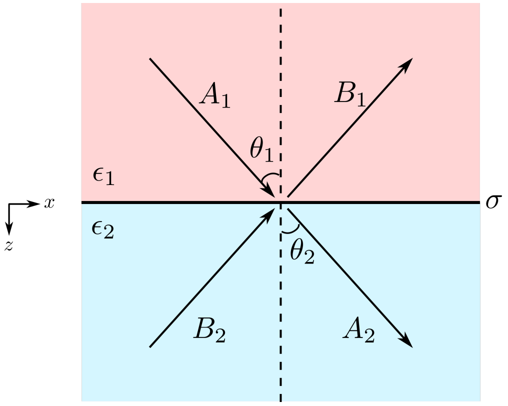

FIG. S1. Scattered fields at a dielectric interface. The boundary has a surface conductivity given by σ.

Consider scattering of an electromagnetic field at the dielectric interface shown in Fig. S1[S1]. For transverse magnetic fields, the magnetic field in each dielectric region is given by

$$\mathbf{B}^{(j)}(\mathbf{r},t) = \left(A_j e^{ik_{j,z}z} + B_j e^{-ik_{j,z}z}\right) e^{i(qx-\omega t)}\hat{\mathbf{y}} \tag{S1}$$

where $k_{j,z}$, q are the z, x components of the wavevector related by $k_{j,z}^2 = \epsilon_j \omega^2/c^2 - q^2$. From Maxwell's equation, $\nabla \times \mathbf{B} = \mu_0\epsilon_0\partial_t\mathbf{E}$, we find the electric field to be

$$\mathbf{E}^{(j)}(\mathbf{r},t) = \frac{k_{j,z}c^2}{\omega\epsilon_j}\left(A_j e^{ik_{j,z}z} - B_j e^{-ik_{j,z}z}\right) e^{i(qx-\omega t)}\hat{\mathbf{x}}. \tag{S2}$$

The fields in the two dielectric regions are connected by the boundary conditions at $z = 0$ given as

$$E_x^{(1)}(z=0) = E_x^{(2)}(z=0) \tag{S3a}$$

$$B_y^{(1)}(z=0) - B_y^{(2)}(z=0) = \mu_0\sigma E_x^{(1)}(z=0). \tag{S3b}$$

By substituting the fields into the boundary conditions, we find that the coefficients A_j, B_j are related by a simple matrix equation:

$$\begin{pmatrix} A_1 \\ B_1 \end{pmatrix} = \frac{1}{2}\begin{pmatrix} 1+\eta+\xi & 1-\eta-\xi \\ 1-\eta+\xi & 1+\eta-\xi \end{pmatrix}\begin{pmatrix} A_2 \\ B_2 \end{pmatrix} \tag{S4}$$

where $\eta = \epsilon_1 k_{2,z}/\epsilon_2 k_{1,z}$ and $\xi = \sigma k_{2,z}/\omega\epsilon_0\epsilon_2$. The matrix in the above equation is the $transfermatrix$ relating the field on either side of the dielectric interface

$$\mathbf{T}^{1\to 2} = \frac{1}{2}\begin{pmatrix} 1+\eta+\xi & 1-\eta-\xi \\ 1-\eta+\xi & 1+\eta-\xi \end{pmatrix}. \tag{S5}$$

The transmission and reflection amplitudes can be read off the transfer matrix as

$$t = \frac{1}{T_{11}^{1\to 2}}, \quad r = \frac{T_{21}^{1\to 2}}{T_{11}^{1\to 2}}. \tag{S6}$$

The transmitted and reflected intensities are then given by

$$T = \frac{\epsilon_1 k_{2,z}}{\epsilon_2 k_{1,z}}|t|^2, \quad R = |r|^2. \tag{S7}$$

In addition to a transfer matrix for a dielectric interface, we also need one for free space propagation. It is simple to see that this matrix is given by

$$\mathbf{P}(d) = \begin{pmatrix} e^{-ik_z d} & 0 \\ 0 & e^{ik_z d} \end{pmatrix}. \tag{S8}$$

Any general layered structure can be completely described as a collection of dielectric interfaces and free space propagation. The total transfer matrix of a general layered structure will thus have the following form

$$\mathbf{T}^{tot} = \mathbf{T}^{1\to 2}\mathbf{P}(d_1)\mathbf{T}^{2\to 3}\mathbf{P}(d_2)\cdots\mathbf{T}^{(N-1)\to N}. \tag{S9}$$

S2. DIELECTRIC CAVITY WITH METAL REFLECTOR

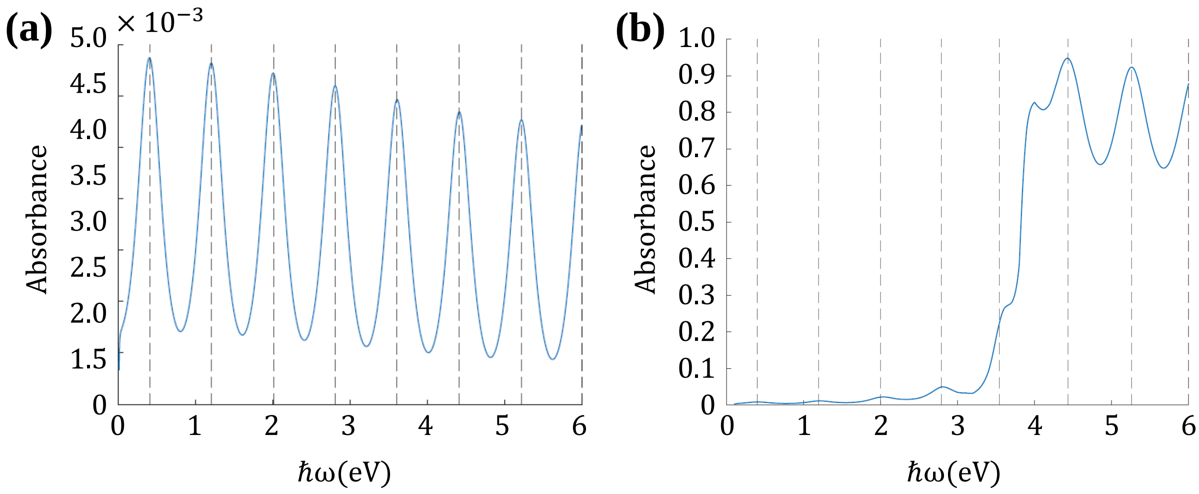

FIG. S2. Absorption of a dielectric cavity with metal reflector. (a) Metal is modeled using the Drude model. (b) Experimental data for silver is used. Vertical dashed lines show the resonant frequencies derived from Eq. (S10)

Consider a three-layer structure where the first layer is air($\epsilon_1 = 1$), the second layer is a dielectric(ϵ_2) of thickness d, and the third layer is a metal(ϵ_3). For such a structure, we can expect resonant absorption when the reflected waves undergo destructive interference. In terms of the dielectric thickness and free space wavelength, the resonance condition can be written as

$$\frac{2d\sqrt{\epsilon_2}}{\lambda} + \frac{\phi_{metal}}{2\pi} = m \tag{S10}$$

where ϕ_{metal} is the phase accumulated upon reflection off the metal and m is an integer. For a perfect conductor $\phi_{metal} = \pi$.

We now calculate the absorption for the setup described in the previous paragraph. The dielectric is set to be SiO_2 with $\epsilon_2 = 2.2$ and thickness $d = 500$nm. The metal is first modeled using the Drude model with $\omega_p = 9$eV and $\gamma = 10$meV. We indeed find resonant absorption determined by the condition derived above (see Fig. S2). Similar results are seen when the metal is defined using experimental data for silver[S2] with the addition of interband absorption from the metal.

S3. ANALYTIC CONDITION FOR PERFECT ABSORBER

Having shown numerically that near perfect absorption can be achieved, we would now like to find an analytic condition on the conductivity for perfect absorption. From the transfer matrix method, it may be shown that the analytic expression for reflection is

$$r = \frac{T_{21}^{1\to2}T_{11}^{2\to3} + T_{22}^{1\to2}T_{21}^{2\to3}e^{i2kd}}{T_{11}^{1\to2}T_{11}^{2\to3} + T_{12}^{1\to2}T_{21}^{2\to3}e^{i2kd}}. \tag{S11}$$

Using the condition for destructive interference, i.e. $e^{i2kd} = -1$, this expression is simplified to

$$r = \frac{\epsilon_2 - \sqrt{\epsilon_3}\left(\sqrt{\epsilon_1} - \sigma/\epsilon_0 c\right)}{\epsilon_2 + \sqrt{\epsilon_3}\left(\sqrt{\epsilon_1} - \sigma/\epsilon_0 c\right)}. \tag{S12}$$

If we assume that the metal is a perfect conductor $\epsilon_3 \to \infty$

$$r \approx -\frac{\sqrt{\epsilon_1} - \sigma/\epsilon_0 c}{\sqrt{\epsilon_1} + \sigma/\epsilon_0 c}. \tag{S13}$$

In general, the conductivity is complex. So by writing $\sigma = \sigma' + i\sigma''$ the reflectivity is given by

$$R = |r|^2 = \frac{\left(\sqrt{\epsilon_1} - \sigma'/\epsilon_0 c\right)^2 + \left(\sigma''/\epsilon_0 c\right)^2}{\left(\sqrt{\epsilon_1} + \sigma'/\epsilon_0 c\right)^2 + \left(\sigma''/\epsilon_0 c\right)^2}. \tag{S14}$$

Hence we may conclude that for the condition $kd = 2\pi(m+1/2)$, the absorption is maximized when $\sigma' = \epsilon_0 c\sqrt{\epsilon_1}$. The maximum of σ' coincides with a zero of σ'' due to the Kramers-Kronig relations which should give perfect absorption. If we assume $\sigma'' \to 0$ and $\epsilon_1 = 1$, the corresponding maximum absorption is simplified as

$$A(\omega) = 1 - \frac{(1 - \sigma'/\epsilon_0 c)^2}{(1 + \sigma'/\epsilon_0 c)^2}, \tag{S15}$$

and the absorbance approaches unity when $\sigma' = \epsilon_0 c$.

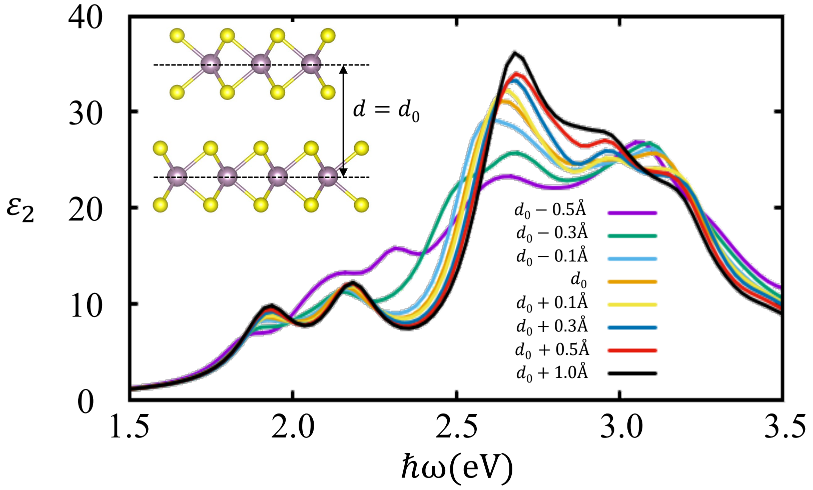

FIG. S3. Imaginary part of dielectric constant (ε_2) of 2L MoS_2 calculated by Bethe-Salpeter equation (BSE) based on GW0 approximation with intentionally manipulated interlayer distance. The intrinsic value of ε_2 was normalized by ration between lattice constant of supercell and thickness of 2L MoS_2.[S3] Here, we used a 15×15×1 k-mesh and a total of 240 bands to obtain converged results. The plane wave cutoff for the response function was chosen to be 266 eV.

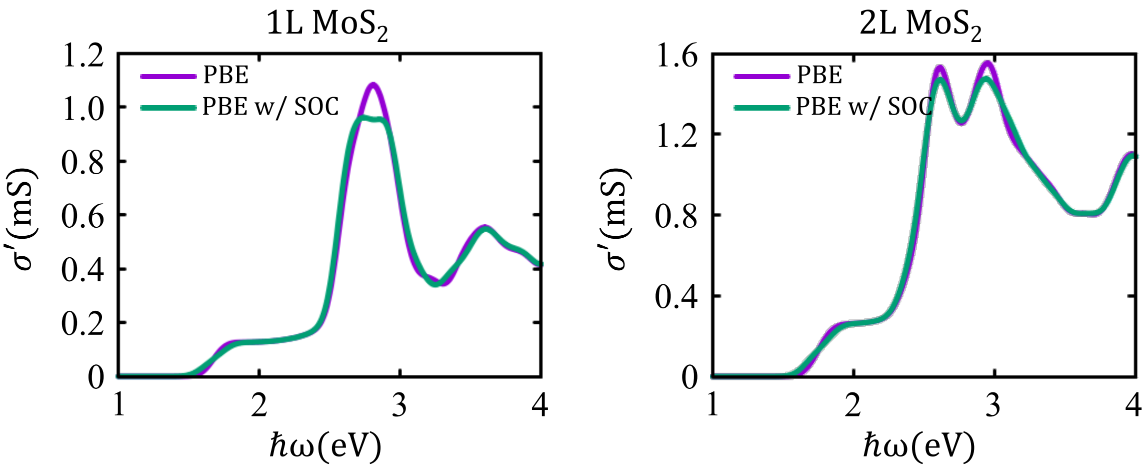

FIG. S4. Spin-orbit coupling dependent real part of 2D optical conductivities of freestanding mono (1L) and bilayer (2L) MoS_2.

S4. LARGE SCALE EXFOLIATION BY AU-ASSISTED METHOD

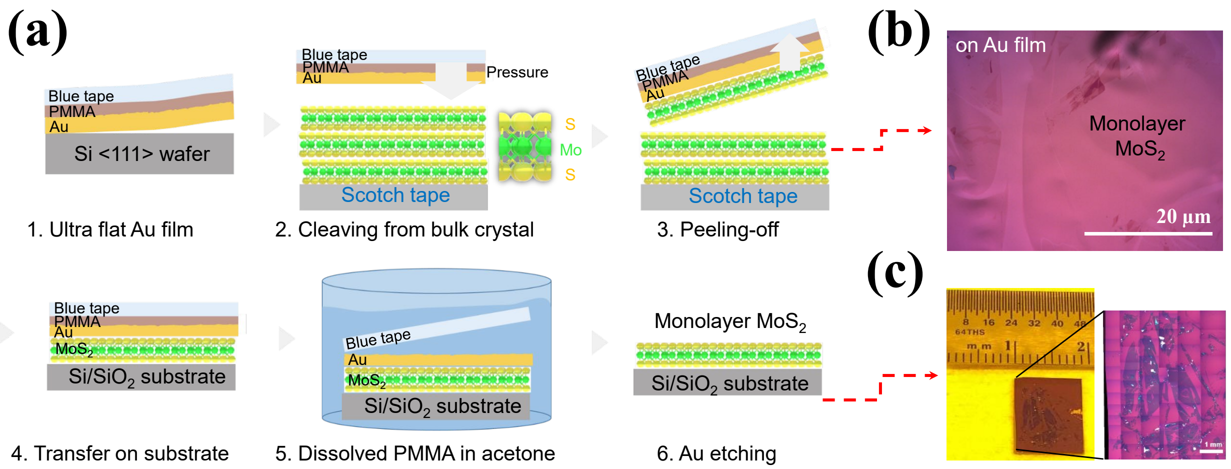

FIG. S5. (a) Schematic illustration of the process of Au-assisted mechanical exfoliation. (b) Optical microscopy image of the large-scale MoS_2 on Au film after the peeling-off process. (c) Optical (left) and microscope (right) image of MoS_2 on Si/SiO_2 substrate.

To overcome the limitations of several micron-sized 2D materials with the commonly used scotch tape method, centimeter-sized monolayer TMD can be obtained using the Au-assisted exfoliation method.[S4,S5] At the first step, a highly polished (111) bare Si wafer was deposited with a 150 nm thick Au film as an ultra-flat template by e-beam evaporation (CHA industries, SEC 600). On top of the Au layer, polymethyl methacrylate (MicroChem, 950 PMMA C4) as protecting layer was spin-coated at a rate of 1000 r.p.m for 60 s. After baking at 120 °C for 2 min, the Au layer is peeling off from the Si substrate with blue tape. Then a freshly cleaved layered bulk MoS_2 was gently pressed on a freshly Au film to establish large-scale MoS_2/Au contact, as shown in Fig. S5 (b). The monolayer MoS_2 is peeled off due to the interaction between Au and the sulfur atom of MoS_2, which is stronger than the interlayer vdW interaction in bulk MoS_2. The MoS_2/Au film/PMMA/blue tape was transferred onto the desired substrate and then dipped in the acetone to dissolve the PMMA to peel off the blue tape. Finally, the top Au film was removed by aqueous KI/I_2 etchant (Sigma Aldrich, "Au etchant, standard") to release the MoS_2.

S5. CHARACTERIZATION OF A LARGE SCALE MoS_2

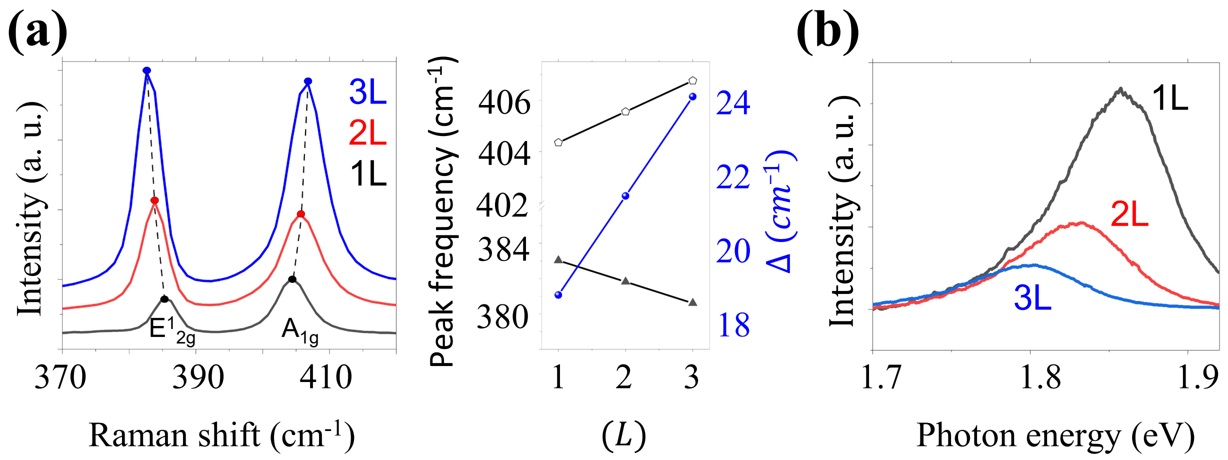

FIG. S6. (a) Raman spectrum for different thicknesses of MoS_2. Frequencies of A_{1g} and E^1_{2g} modes against layer thickness. (b) Photoluminescence (PL) spectra from monolayer to trilayer of MoS_2.

To characterize the MoS_2 by Au film-assisted method, Fig. S6 (a) shows the Raman spectra with different thicknesses of MoS_2. The finger print region in the Raman spectrum of 1L MoS_2 exhibits two main modes E^1_{2g} at 385 cm^{-1} and A_{1g} at 404 cm^{-1} with mode difference of 19 cm^{-1}. This clearly proves that it is monolayer MoS_2, which is the same result as the commonly used scotch tape method.[S6] It successfully reproduces the experimentally observed continuous red-shift E^1_{2g} and blue-shift A_{1g} with increased thickness, as shown by a dashed line. The frequency difference monotonically increases from 19 to 24 cm^{-1} with increasing thickness in the right panel of Fig. S6 (a), confirming the frequency difference as a reliable thickness indicator. The indirect-to-direct band gap crossover in 1L is a common property of all the semiconducting TMD investigated so far. Fig. S6 (b) shows room-temperature PL spectra at 532 nm excitation with different thicknesses of MoS_2. The characteristic PL emission can be observed in 1L MoS_2 at 1.86 eV, corresponding to a direct transition at the band edge of κ point. As the number of layers increases, the PL emission peaks are red-shift at 1.83 (2L) and 1.79 eV (3L), respectively.

S6. EXEMPLARY SPECTRUM FOR OPTICAL CONTRAST

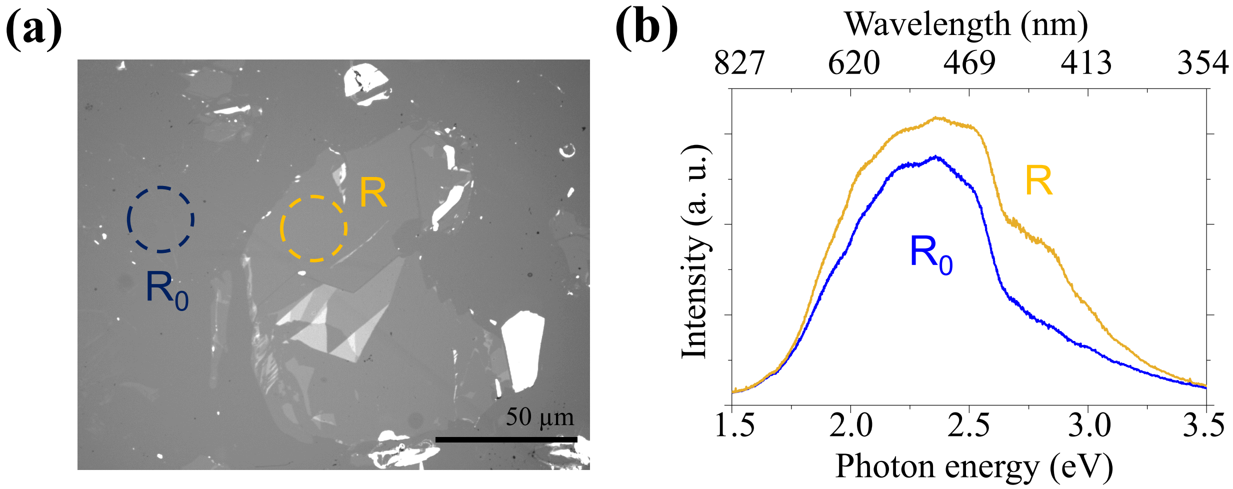

FIG. S7. (a) Optical microscope image of a MoS_2 on glass. (b) The reflection spectra of 1L on glass and Only the glass area has been highlighted with a dashed orange and blue circle in (a), respectively.

Fig. S7(a) shows a reflection mode optical image of a MoS_2 on a glass substrate by Au-assisted exfoliation method. Fig. S7(b) shows the reflectance spectra acquired at the positions marked in the optical image of Fig. S7(a) with an orange and blue dashed circle. Optical contrast was calculated as (R-R_0)/R_0, and is directly proportional to the absorbance of the film,[S7,S8]

$$\frac{R - R_0}{R_0} = \frac{4n}{n_0^2 - 1}\alpha(\lambda), \tag{S16}$$

where R is the reflection spectra of the MoS_2 on the substrate, R_0 is the reflection spectra of the substrate, n is the refractive index of MoS_2, and n_0 is the refractive index of the substrate.

S7. FABRICATION AND OPTICAL CONTRAST OF TWIST 2L MoS_2

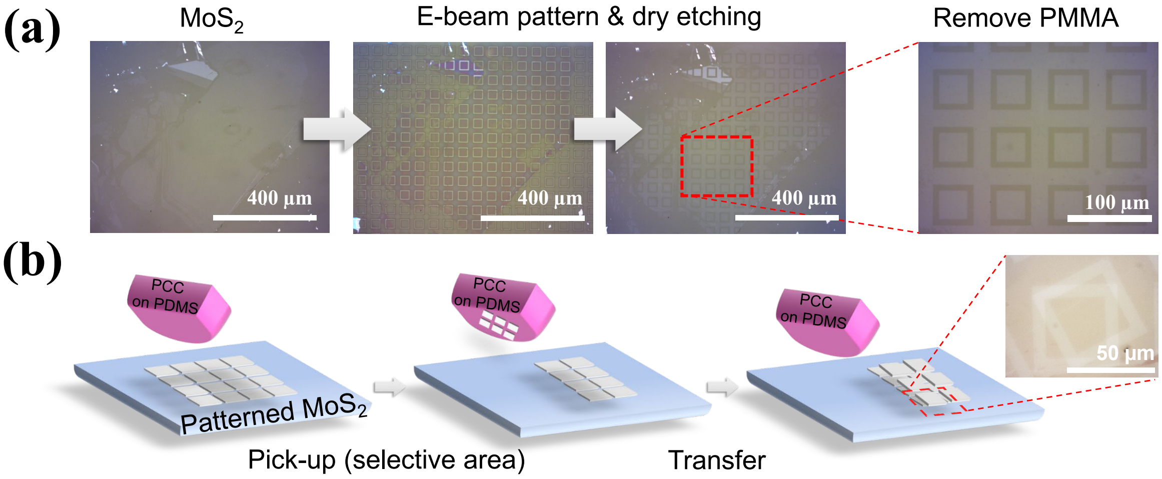

FIG. S8. (a) Fabrication process of twisted 2L MoS_2. (b) Schematic image of the pick-up and transfer method by a poly-propylene carbonate (PCC) polymer. This process takes place within a single crystal MoS_2.

In order to support the hypothesis that the interlayer coupling contributes to the absorption with different twist angles, a control sample of twist 2L MoS_2 is assembled using standard dry-transfer techniques with a poly-propylene carbonate (PCC) stamp. As shown in Fig. S8 (a), fabrication began with the large-scale MoS_2 onto glass substrate by Au-assisted exfoliation method. An electron-beam resist, polymethyl methacrylate (MicroChem, 950 PMMA C4), was spin-coated at a rate of 3,000 r.p.m for 1 min. After baking at 180 °, AquaSave, a water-soluble conductive polymer, was spin-coated at a rate of 1,000 r.p.m for 1 min without baking. The PMMA/AquaSave was exposed to electron beams at 100 keV energy with an exposure dose of 650 $\mu C/cm^2$ using an electron-beam lithography system (Vistec, EBP5000+). AquaSave was dissolved with DI water for 1 min and then developed with a solution of MIBK:IPA (1:3) for 60 s to generate a PMMA square pattern. Using PMMA pattern array as a mask, MoS_2 was etched using a reactive ion etcher (Advanced Vacuum, Vision 320). The PMMA etch mask was removed with Acetone. The twist angle can be determined by the relative orientation of the top and bottom square shapes.

Fig. S8 (b) shows a schematic image of twist 2L MoS_2 preparation. We utilized dry transfer procedures[S9] with polypropylene carbonate (PPC) to fabricate twist 2L MoS_2. The

PPC film was placed on the poly (dimethyl siloxane) (PDMS)/glass slide. By controlling the temperature on a hot plate, patterned MoS_2 in the selective area was picked up. Then, by controlling the rotation angle, patterned MoS_2 was released onto nearby patterned MoS_2 with the same single crystal. Finally, PPC film was dissolved in Chloroform.

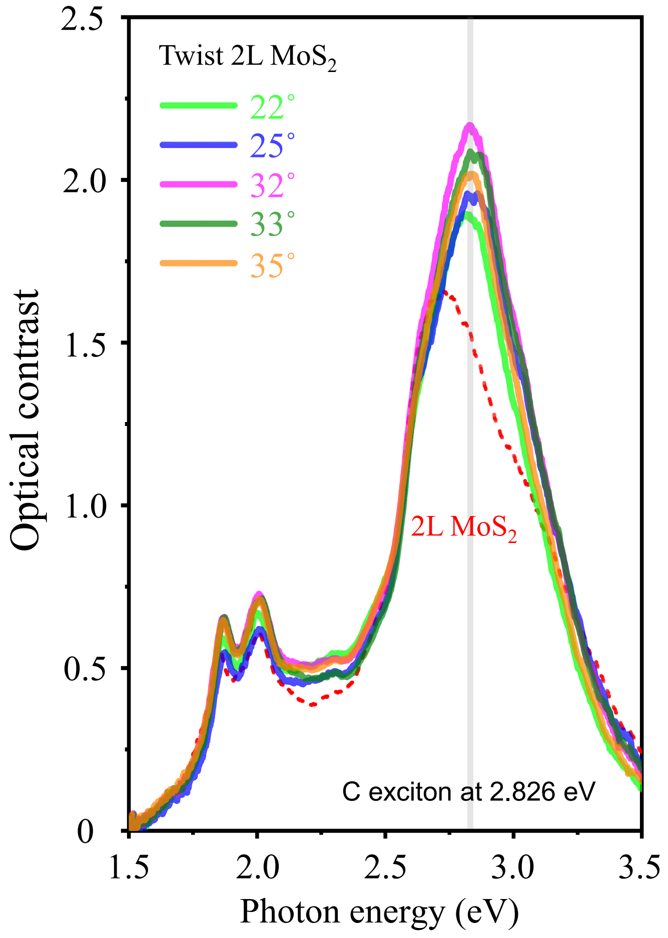

FIG. S9. Optical contrast of twist 2L MoS_2 with precise-controlled twist angles from 22 to 35°. The red dashed line represents the natural bilayer MoS_2.

To characterize these samples, Fig. S9 (a) shows the optical contrast of 2L MoS_2 with precise-controlled twist angles from 22 to 35°. Optical contrast has two prominent narrow peaks, associated with the generation of the A and B excitons, respectively. And the spectra also show a broad feature around 2.83 eV, referred to as the C excitons peak. At the twist angle of 32°, which is very close to the designed twist angle of 30°. At this point, the intensity of the C exciton at twist angle of 32° can reach the highest value compared to that of other

twist angles. We confirm the gradual increase and decrease of the C exciton peak intensity in the center of θ=30°. Compared to the natural 2L MoS_2(red dashed line), it shows a stark angle dependence of the C exciton, in very good agreement with our theoretical calculation. This is caused by interlayer coupling according to the interlayer distance, which leads to the energy shift of a critical point between Γ and Q valleys. Also, a greater tunability in the interlayer distance by twist angle results in a higher light absorption near the band-nesting region. We emphasize the similarity between the optical contrast of c exciton and interlayer distance on the twist angle.

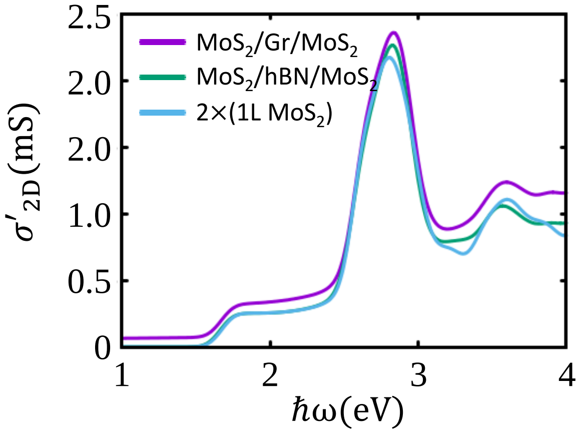

FIG. S10. Calculated real part of 2D optical conductivities of MoS_2/graphene/MoS_2 (purple) and MoS_2/hBN/MoS_2 (green) heterostructures. Skyblue solid line shows two times the 2D optical conductivity of MoS_2.

S8. WEAK INTERFACIAL ELECTRONIC COUPLING OF MoS_2/Gr/MoS_2 HETEROSTRUCTURE

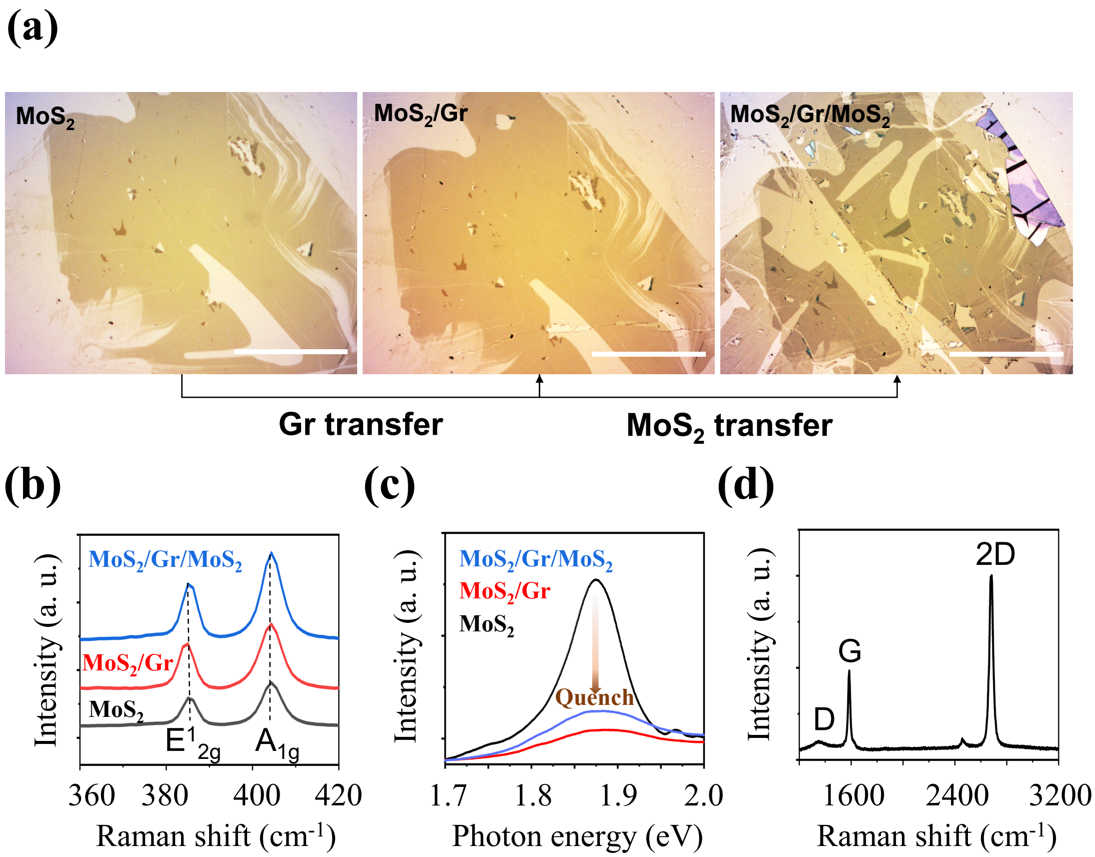

FIG. S11. (a) Microscope image of sample preparation of stacked MoS_2/Gr/MoS_2 heterostructure with 100 µm scale bar. (b) and (c) Local Raman and PL spectra of MoS2 for each step.(d) Raman spectra of Gr.

Fig. S11 shows the preparation process of MoS_2/Gr/MoS_2 heterostructure. After getting a large scale MoS_2 by Au-assisted exfoliation method, the chemical vapor deposition (CVD)-grown graphene films were transferred onto the MoS_2 film. The following process pertains to the transfer of CVD-grown graphene on copper(Cu) foil. PMMA(MicroChem, 950 PMMA C4) was spin-coated on CVD-grown Gr on Cu foil at 3,000 r.p.m for 1 min and baked at 180 °C for 15 min. After the back side graphene was removed by Oxygen plasma cleaning for 1 min, the sample was floated on Ammonium persulfate solution (7g/1L). After etching the Cu foil, graphene/PMMA was transferred to float on DI water for 10 min, 3 times. The

graphene/PMMA film was transferred to the MoS_2 film and baked at 180 °C for 30 min. PMMA was removed with Acetone. Finally, MoS_2 by Au-assisted exfoliation method was picked up and transferred by dry transfer method.

In Fig. S11(b) shows the Raman spectra of MoS_2 region (360-420 cm^{-1}). In Fig. S11 (b), we observed the two Raman modes at approximately 383.7 and 402.1 cm^{-1} of 1L MoS_2, corresponding to the E^1_{2g} and A_{1g} modes. The E^1_{2g} and A_{1g} modes after transferring graphene were almost identical compared to those of 1L MoS_2. We confirmed the same trend from stacked top MoS_2, regardless of the stacking angle. The Raman results show that the doping or strain of MoS_2 by graphene has a negligible effect on the shift of E^1_{2g} and A_{1g} modes. Fig. S11 (c) shows PL measurement of MoS_2 in heterostructure, which reveals significant quenching of over 50 % in intensity when compared to 1L MoS_2. This is usually ascribed to charge transfer process. The considerable PL quenching can be attributed to the reduced recombination of e–h pairs through the heterojunction of graphene/MoS2. However, as shown in Fig. S12, it does not affect the light absorption of MoS_2. Fig. S11(d) show Raman with c-c bonds of graphene: D, G, and 2D peak. The ratio of I_{2D}/I_G is about 1.58, close to 2 of high-quality single-layer graphene.

To investigate the effect of graphene used as a buffer layer, we performed the optical contrast and absorbance with a comparison between 1L MoS_2 and MoS_2/Gr heterostructure. Fig. S12 (a) shows the optical contrast of intrinsic (1L(black line) and 2L(blue line)) MoS_2 and MoS_2/Gr heterostructure(red line). Increasing the number of layers from 1L to 2L of MoS_2, the energy of A exciton was slightly redshift due to the interlayer coupling. The B exciton, on the other hand, shows a slight blueshift. Interestingly, the C exciton peak also shifts and its dependence is even stronger than that of A and B exciton. In the case of MoS_2/Gr heterostructure, all of the exciton (A, B, and C) position holds the same positions as those of 1L MoS_2. Also, Fig. S12 (b) shows the absorbance of intrinsic (1L and 2L) MoS_2 and MoS_2/Gr heterostructure. We confirmed that the absorbance of the MoS_2/Gr heterostructure(red line) without exciton peak shift increases by 2.2 % compared to that of 1L MoS_2(black line). In other words, it is clear evidence that graphene has no effect on the exciton absorbance of MoS_2.

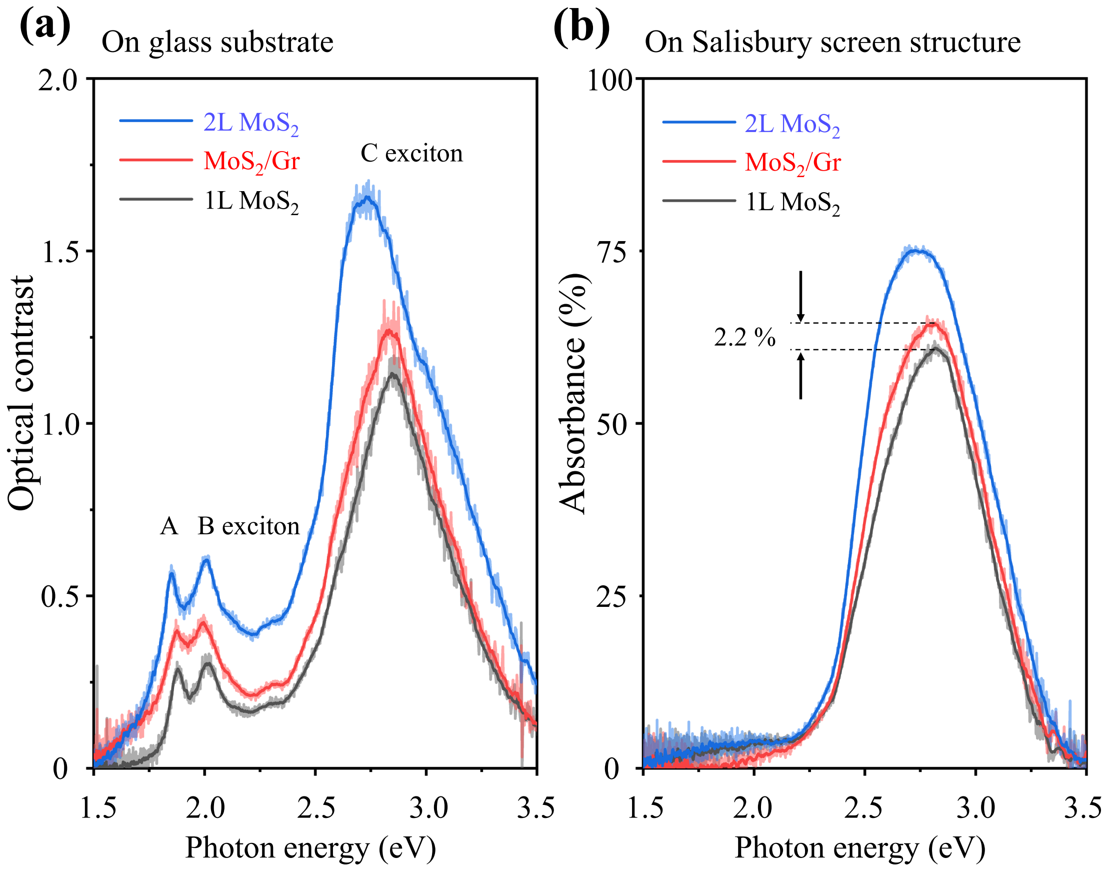

FIG. S12. (a) Optical contrast (b) absorbance of intrinsic (1L and 2L) MoS_2 and MoS_2/Gr heterostructure on glass and Salisbury screen, respectively.

S9. MOLECULAR BEAM EPITAXY GROWTH

The WSe_2/ZnSe/WSe_2 heterostructure growth was performed on a double side polished (DSP) sapphire (0001) wafers using molecular beam epitaxy (MBE). The sapphire was first degassed at 900°C for 60 min and then ramped down to the growth temperature of 600°C using a ramp rate of 30°C/min. The growth process starts with the deposition of the bottom WSe_2 monolayer (1L) by co-depositing tungsten (W), evaporated using a multi-pocket e-beam evaporator, and elemental selenium (Se), evaporated using a cracker source. A tungsten to selenium flux ratio of 1:200 was used. The W flux ($\sim 5 \times 10^{-9}$ mbar) was obtained using an e-beam current of 200 mA and voltage of 6 kV while the Se flux (1×10^{-6} mbar) was controlled by setting the temperature of the Se reservoir to 130 °C and its cracker tip to 1100 °C. The WSe_2 growth rate was 6 hours/1L. The 1L coverage was confirmed by the disappearance of the reflection high energy electron diffraction (RHEED) pattern of the sapphire substrate and the concomitant appearance of a new RHEED pattern corresponding to the WSe_2. The buffer layer growth of 1 nm ZnSe was grown at 600 °C in the same chamber with the zinc (cell temperature: 320°C, flux: 2×10^{-6} mbar) and selenium (cell temperature: 190°C, flux: 2×10^{-7} mbar) co-evaporated using low-temperature Knudsen cells. A zinc to selenium flux ratio of 10:1 was used. The top 1L of WSe_2 was then grown at 600°C on this ZnSe template using the same W e-beam evaporator and Se cracker source. No post-growth annealing was performed to avoid any high temperature degradation of the bottom layers. To make the optical cavity, the sample was taken out of the MBE, moved to a plasma enhanced chemical vapor deposition (PECVD) chamber, and capped with 192 nm of SiO_2 deposited at 250°C. Here, thickness of SiO_2 was chosen to be 192 nm, satisfying the critical coupling condition for the photon energy of 2.92 eV of B' exciton of WSe_2. Lastly, 3 nm of titanium followed by 100 nm of silver was deposited using e-beam evaporation to be used as the back reflector.

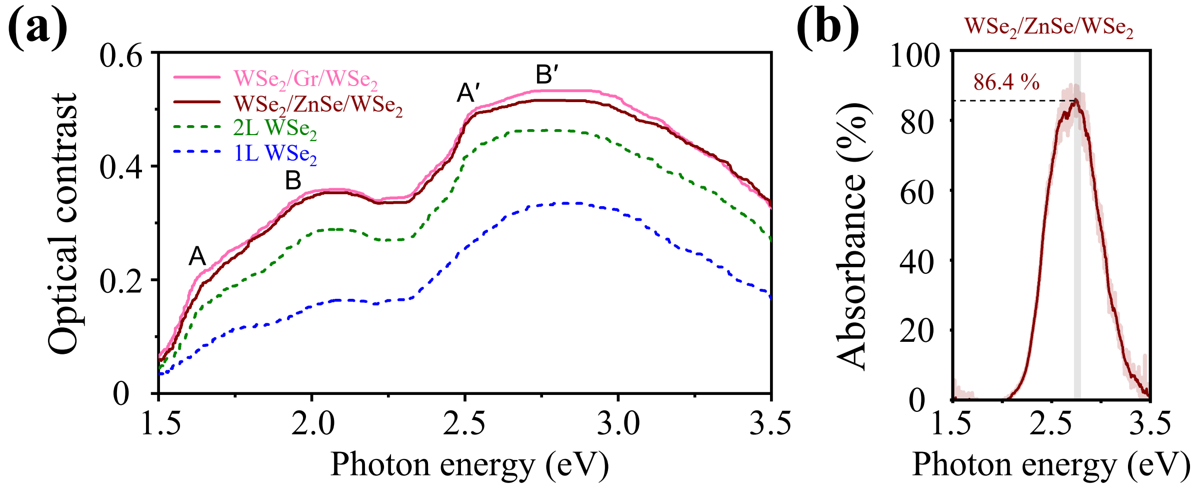

FIG. S13. Optical contrast of molecule beam epitaxy (MBE) grown WSe_2 heterostructure (a) on sapphire and (b) on Salisbury screen structure with 192 nm thickness of SiO_2 as a dielectric spacer and a silver reflector.

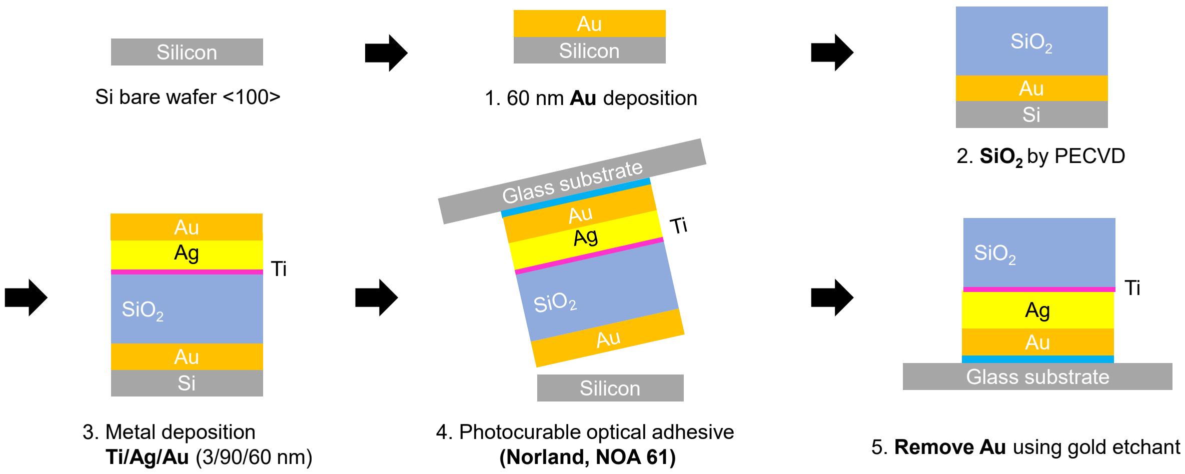

FIG. S14. Fabrication process; Before the fabrication, a bare silicon wafer was first heated on a hot plate at 180°C for 5 min and then surface treatment by oxygen plasma (Advanced vacuum, Vision 320) for 5 min. 1. Gold (Au) deposition with a 60 nm thick as a sacrificial layer by an electron-beam evaporator (CHA industries, SEC 600). 2. SiO_2 deposition by plasma-enhanced chemical vapor deposition (Plasma-Therm, PECVD). 3. Silver (Ag) and Au deposition at 90 nm and 60 nm thick, respectively, by an electron-beam evaporator (CHA industries, SEC 600). 4. Peeling off the whole structure (template stripping method) using Photocurable epoxy (Norland, NOA 61) as an adhesive to transfer the entire cavity structure to a glass substrate. 5. Remove the sacrificial layer of the top gold film with a gold etchant (Sigma Aldrich).

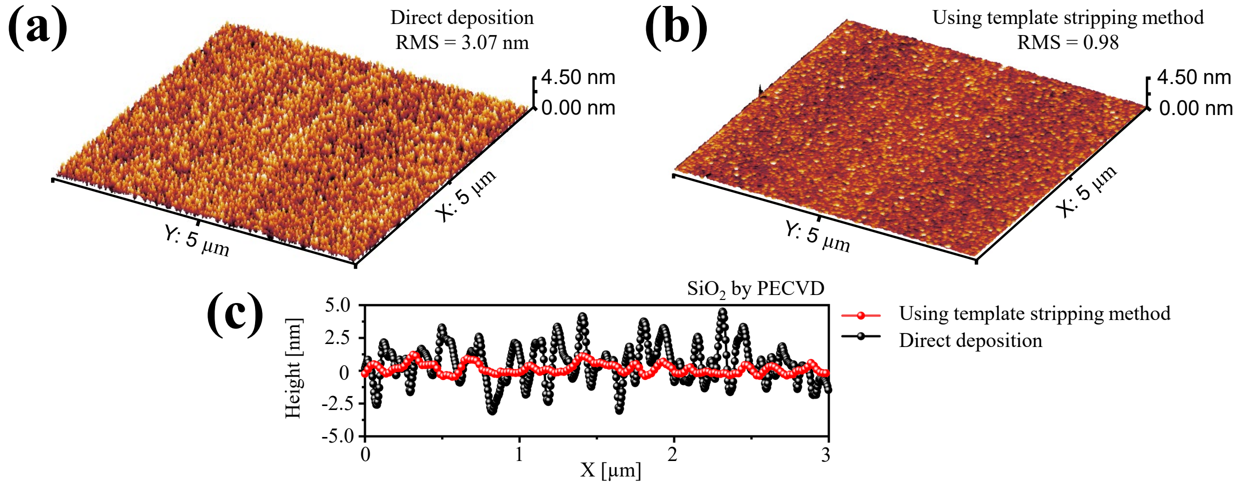

FIG. S15. (a) AFM images of (a) SiO_2 on Si substrate and (b) SiO_2 using template stripping method. (c) Line profile of cross section of SiO_2.

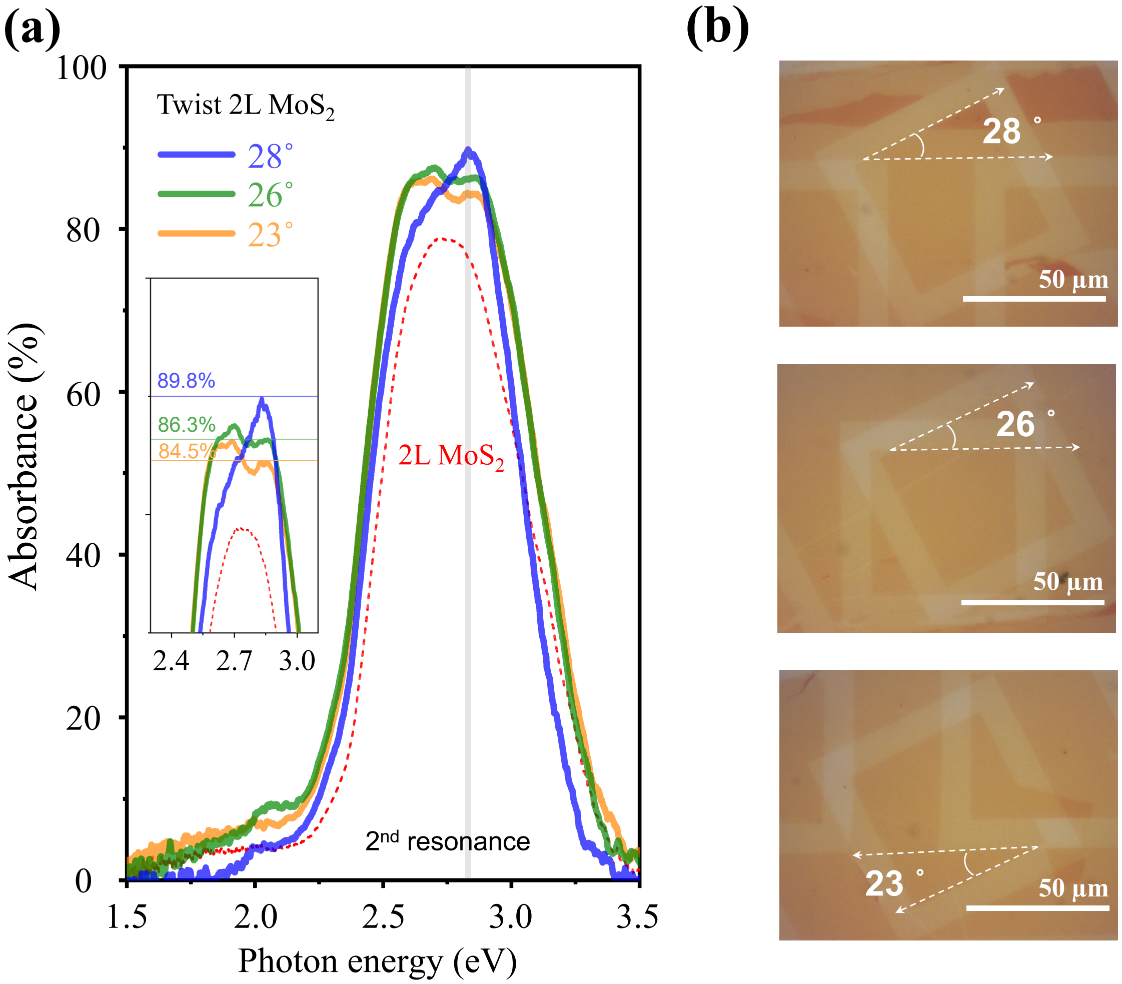

FIG. S16. (a) Absorbance and (b) microscope images of twisted 2L MoS_2 on Salisbury screen structure with rotation angles of 28, 26, and 23 °

S10. ABSORBANCE OF FEW-LAYER MoS_2

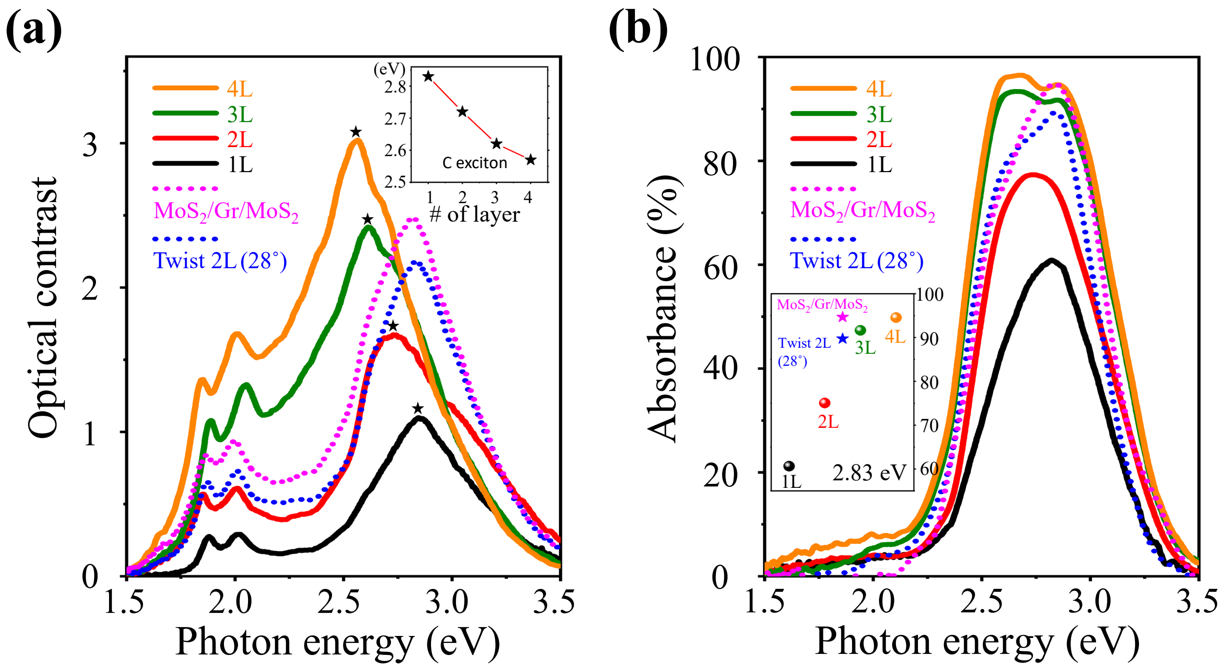

FIG. S17. (a) Optical contrast of few-layer MoS2 on a glass substrate and (b) absorbance with Salisbury screen. Insets in (a) and (b) indicate a degree of the redshift of C exciton and maximum absorbance with Salisbury screen, respectively.

Figure S17(a) and (b) show the absorbance of 3L and 4L of MoS_2 on a glass substrate and with Salisbury screen, respectively. As MoS_2 becomes thicker, the optical contrast continues to increase with gradual redshift, all of which are in good agreement with previous studies.[S10,S11] The maximum optical contrast of twisted 2L MoS_2 and MoS_2/Gr/MoS_2 heterostructure is almost the same as that of 3L MoS_2 with negligible redshift. Note that such a negligible redshift is practically useful in designing Salisbury screen since its optimum thickness of dielectric layer is the same as that of the monolayer case. We don't have experimental results on twisted 3L and 4L of MoS_2. However, it is naturally deduced that twisted 3L MoS_2 should have higher optical contrast (or real part optical conductivity) than pristine 3L MoS_2, and also should exhibit much weaker redshift.

With the Salisbury screen structure, both 3L and 4L of MoS_2 also exhibit very strong absorbance because their optical conductivity is also very close to the near-perfect light absorbers (NPLAs) condition. (2.17 mS $< \sigma' <$ 3.24 mS) Our DFT results (Fig. S18(a) and

(b)) also exhibit consistent results with experiments. It can be particularly useful to utilize twisted 3 or 4 layers to design "perfect" light absorbers ($\mathcal{A} > 99.9\%$ or even higher). The perfect light absorber with Salisbury screen requires a specific value of σ' very close to 2.654 mS, which is not feasible just using pristine few-layer TMDs. The close relation between twist angle and band nesting of TMDs opens a feasible pathway to design a perfect light absorber, providing a new perspective on efficient atomically thin optoelectronics.

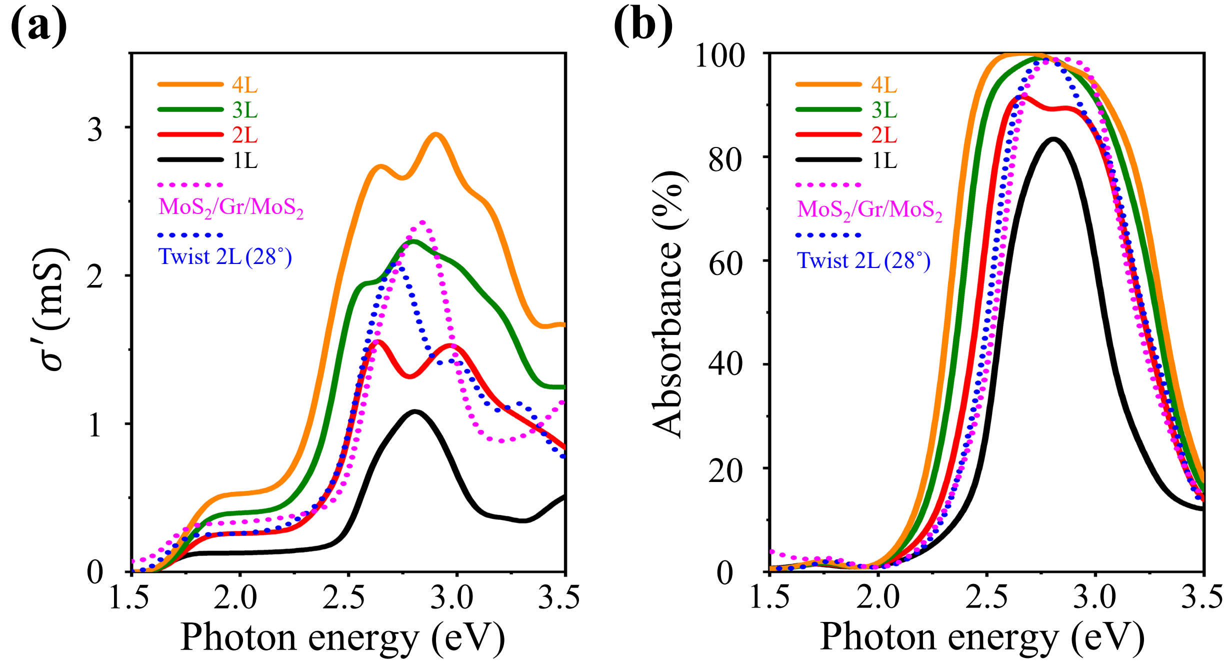

FIG. S18. (a) Theoretically calculated real part of optical conductivities and (b) absorbance with Salisbury screen of few-layer MoS2.

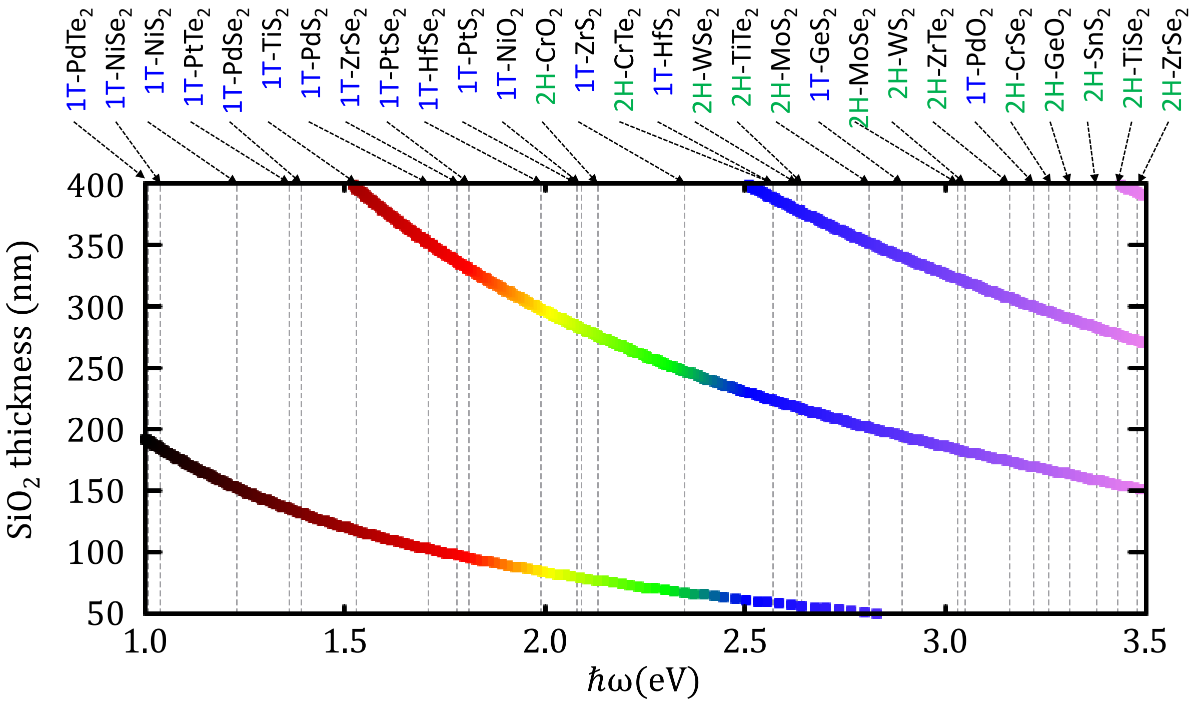

FIG. S19. The wavelength-dependent optimum thickness of SiO_2 satisfying a resonance condition on the Ag mirror reflector. The optimum wavelength of various TMD materials were also represented in the dashed lines. For example, 1L MoS_2 theoretically exhibits the highest absorbance at 2.81 eV, with the corresponding multiple optimum SiO_2 thicknesses of 51, 201, and 351 nm.

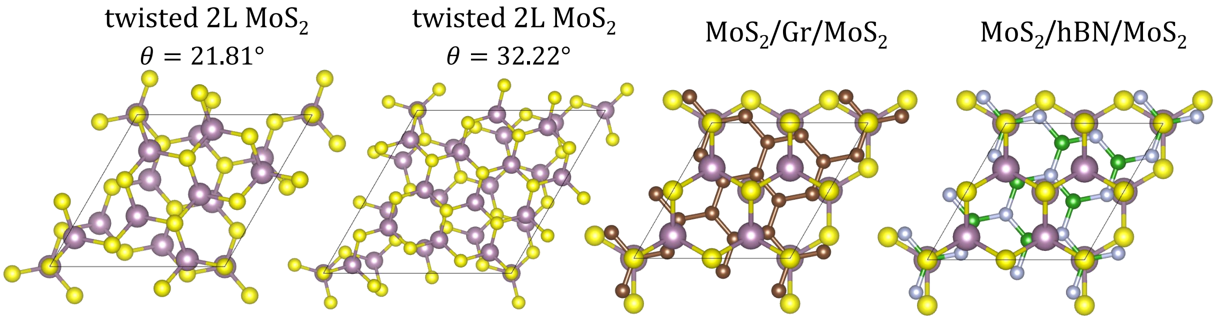

FIG. S20. Supercell structures of twisted 2L MoS_2 and MoS_2/buffer layer/MoS_2 heterostructures, which were used in the DFT calculations. Here, the selected twist angles (21.81 and 32.22°) allow commensurate supercells (zero-strain) in DFT calculations. With the buffer layer, we used a 2×2 supercell of MoS_2 and the artificial strains were only applied to the buffer layer.

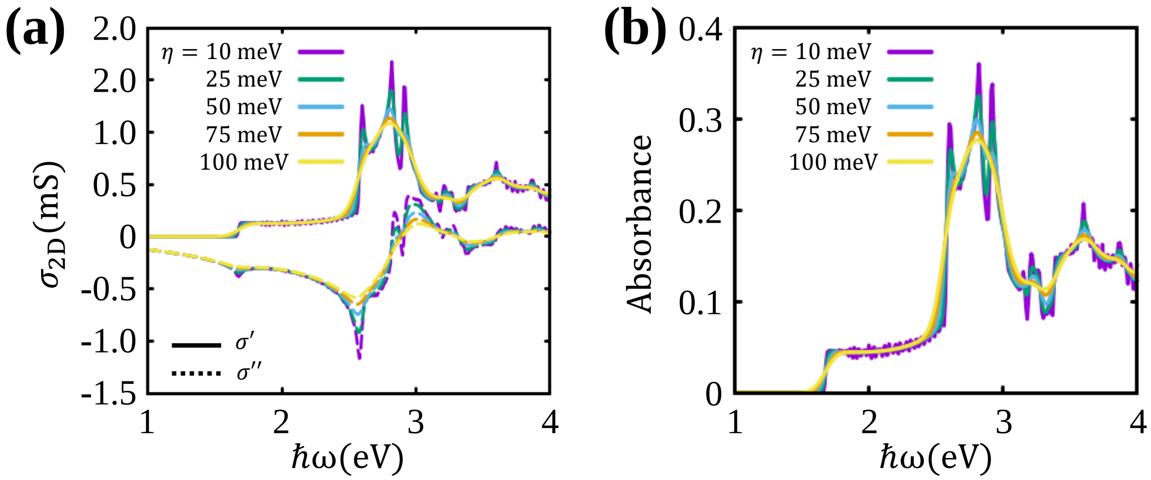

FIG. S21. (a) 2D optical conductivity and (b) absorbance of 1L MoS_2 calculated by Kubo formula with various value of η.

* These authors contributed equally to this work.

† skoester@umn.edu

‡ tlow@umn.edu

[S1] Goncalves, P. & Peres, N. M. *An Introduction to Graphene Plasmonics* (World Scientific, 2016).

[S2] Babar, S. & Weaver, J. H. Optical constants of Cu, Ag, and Au revisited. *Appl. Opt.* **54**, 477–481 (2015).

[S3] Yang, G. & Gao, S.-P. A method to restore the intrinsic dielectric functions of 2d materials in periodic calculations. *Nanoscale* **13**, 17057–17067 (2021).

[S4] Huang, Y. *et al.* Universal mechanical exfoliation of large-area 2D crystals. *Nature Commun.* **11**, 2453 (2020).

[S5] Liu, F. *et al.* Disassembling 2D van der Waals crystals into macroscopic monolayers and reassembling into artificial lattices. *Science* **367**, 903–906 (2020).

[S6] Lee, C. *et al.* Anomalous lattice vibrations of single-and few-layer MoS_2. *ACS nano* **4**, 2695–2700 (2010).

[S7] McIntyre, J. & Aspnes, D. E. Differential reflection spectroscopy of very thin surface films. *Surf. Sci.* **24**, 417–434 (1971).

[S8] Dhakal, K. P. *et al.* Confocal absorption spectral imaging of MoS_2: optical transitions depending on the atomic thickness of intrinsic and chemically doped MoS_2. *Nanoscale* **6**, 13028–13035 (2014).

[S9] Wang, L. *et al.* One-dimensional electrical contact to a two-dimensional material. *Science* **342**, 614–617 (2013).

[S10] Castellanos-Gomez, A., Quereda, J., van der Meulen, H. P., Agraït, N. & Rubio-Bollinger, G. Spatially resolved optical absorption spectroscopy of single- and few-layer MoS_2 by hyperspectral imaging. *Nanotechnology* **27**, 115705 (2016).

[S11] Neri, I., López-Suárez, M., Caponi, S. & Mattarelli, M. Fast MoS_2 thickness identification by transmission imaging. *Appl. Nanosci.* **11**, 605–610 (2021).
